# Iatrogenic Sciatic Nerve Injury Following Surgical Treatment of an Acetabular Fracture: A Case Report of Conservative Management

**DOI:** 10.7759/cureus.108545

**Published:** 2026-05-09

**Authors:** Amal Aoussaf, Sarra Elhamlili, Siham Elmir

**Affiliations:** 1 Physical Medicine and Rehabilitation, Faculty of Medicine and Pharmacy, Mohammed I University, Oujda, MAR

**Keywords:** electromyography (emg), foot drop, iatrogenic nerve injury, platelet-rich plasma (prp), rehabilitation therapy, sciatic nerve injury

## Abstract

Iatrogenic sciatic nerve injury is a recognized but uncommon complication of acetabular fracture surgery, often leading to significant functional impairment. We report the case of a 20-year-old man who was hit by a car and sustained a left hip dislocation with an ipsilateral acetabular fracture and developed iatrogenic sciatic nerve injury with foot drop following open reduction and internal fixation of the acetabular fracture. Electrodiagnostic studies confirmed predominant peroneal division involvement with denervation changes on needle electromyography. The patient underwent a comprehensive rehabilitation program combining orthotic management, physical therapy, and ultrasound-guided platelet-rich plasma injections around the sciatic nerve and the ankle dorsiflexor muscles. Notable functional improvement was observed within six months . This case highlights the importance of early electrodiagnostic evaluation, multimodal rehabilitation, and the possible role of platelet-rich plasma as an adjunct therapy in the management of iatrogenic sciatic nerve injury following acetabular fracture surgery.

## Introduction

Sciatic nerve injury is one of the potential complications of acetabular fractures, which can occur either because of the initial trauma or during the surgical reconstruction, or as a delayed postoperative complication [1]. The prevalence of sciatic nerve injury associated with acetabular fractures ranges from 10% to 30% [1]. However, the reported rate of iatrogenic sciatic nerve injury following acetabular fracture surgery ranges from 5% to 15% [1].

Although a significant proportion of sciatic nerve injuries recover spontaneously, the prognosis is variable and depends on the severity and mechanism of the lesion, the division affected, and the time elapsed before treatment [2], with electrodiagnostic studies used to further assess these prognostic factors. Foot drop is a neuromuscular condition characterized by weakness or paralysis of the dorsiflexor muscles, which causes difficulty lifting the forefoot during gait and results in a high-steppage walking pattern. Emerging adjunctive therapies, such as platelet-rich plasma (PRP), have been proposed for the treatment of peripheral nerve injuries, although current evidence remains limited.

When recovery is incomplete, patients may suffer from lasting motor and sensory deficits, chronic neuropathic pain, and severe functional impairment, with major repercussions on quality of life [3]. We report a case of iatrogenic sciatic nerve injury with foot drop following fracture-dislocation of the hip in a young patient.

## Case presentation

**Figure 1:**
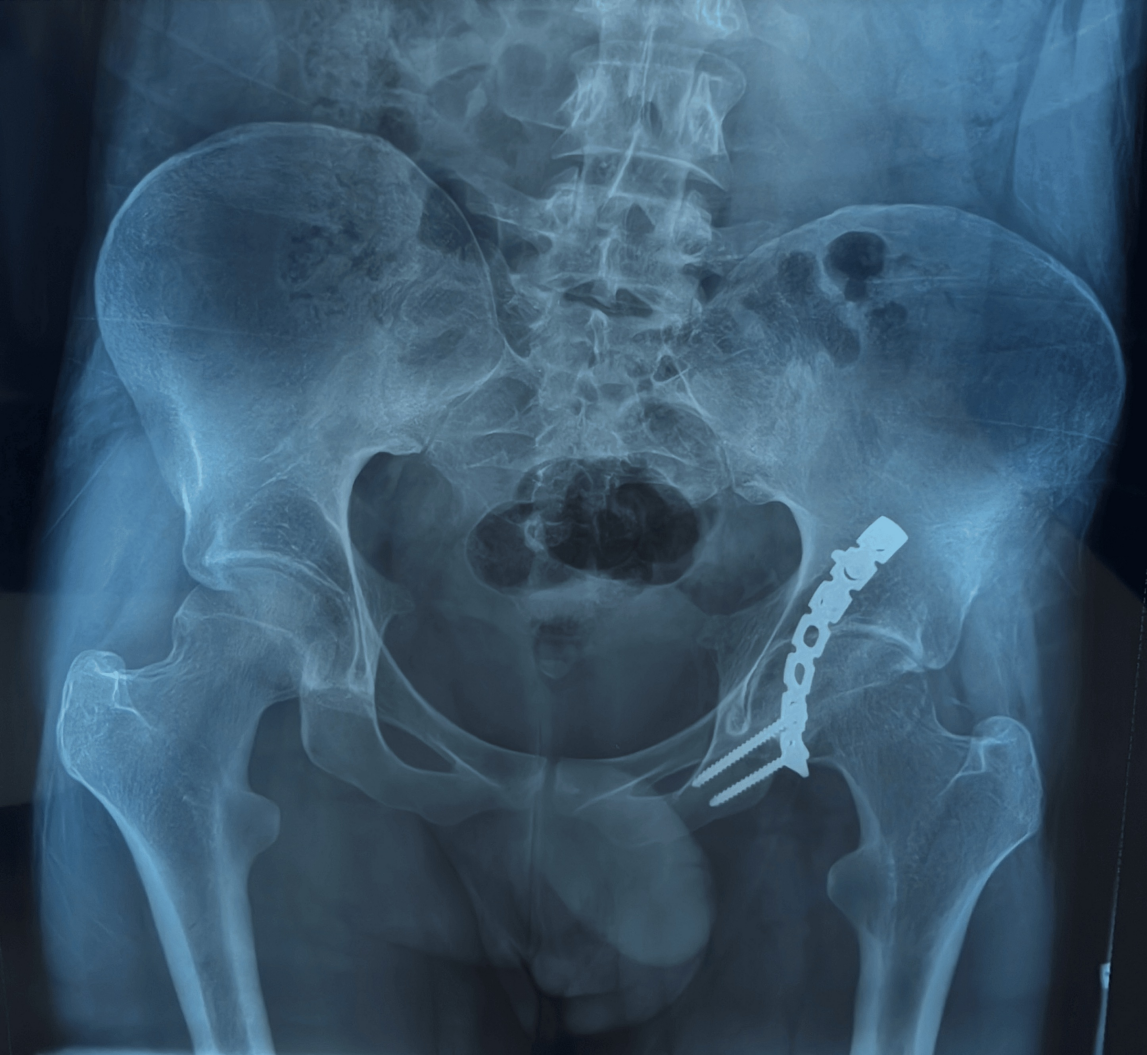
Postoperative pelvic X-ray showing plate and screw fixation of a posterior wall acetabular fracture. The posterior approach places the sciatic nerve in close proximity to the surgical field, highlighting the risk of iatrogenic sciatic nerve injury in this anatomical context.

A 20-year-old man with no past medical history was hit by a car three months before his admission to our department. The initial assessment at the emergency department revealed a left hip dislocation associated with an ipsilateral acetabular fracture. The hip dislocation was promptly reduced. The patient was then transferred to the operating room and underwent open reduction and internal fixation (ORIF) of the acetabular fracture via a posterior approach (Figure 1). 

Three months later, the patient was referred to our Department of Physical Medicine and Rehabilitation. On admission to our department, neurological examination of the left lower limb revealed significant motor weakness. Muscle strength, assessed using the Medical Research Council (MRC) grading scale, demonstrated hip abduction graded at 3/5 and hip adduction graded at 5/5, as well as knee flexor strength of 3/5. Severe impairment of ankle dorsiflexors and hallux extensor at 1/5, and foot eversion at 1/5, with foot inversion graded at 3/5, and relatively preserved ankle plantar flexion at 4/5 [4]. Deep tendon reflexes of the left lower limb were diminished. Sensory examination revealed hypoesthesia along the lateral aspect of the left lower leg and the dorsum of the foot, with anesthesia over the plantar surface. The patient exhibited a characteristic high-stepping gait. Examination of the lumbar spine was unremarkable; however, Lasegue's sign was positive on the left, and palpation of the gluteal region elicited pain.

Electrodiagnostic studies were subsequently performed. Nerve conduction studies revealed normal left tibial motor amplitude but absent left sural sensory response, and absent motor responses from both superficial and deep fibular nerves. F-wave latencies of the tibial nerve were within normal limits, while those of the peroneal nerve could not be obtained. Needle electromyography (EMG) demonstrated active denervation with nascent reinnervation potentials in the long head of the biceps femoris, tibialis anterior, extensor hallucis longus, extensor digitorum brevis, and medial gastrocnemius. The quadriceps and lumbar paraspinal muscles were normal.

Based on the clinical presentation, operative history, and electrodiagnostic findings, a diagnosis of left sciatic nerve injury was established, with predominant involvement of the peroneal division resulting in foot drop.

A comprehensive rehabilitation protocol was initiated, including orthotic management, physical therapy, and neuromuscular stimulation. The main goals were to prevent secondary complications such as equinus contracture and muscle atrophy, maintain joint mobility and muscle strength, and promote functional recovery. Gait training and a structured home exercise program were progressively introduced to improve independent ambulation and ensure continuity of rehabilitation (Table 1).

**Table 1:** Comprehensive rehabilitation protocol.

Component	Intervention	Objective
Orthotic management	Custom-fitted ankle-foot orthosis (AFO)	Maintain neutral ankle position, prevent equinus deformity, improve foot clearance during gait, reduce fall risk
Range of motion exercises	Passive and active-assisted exercises for ankle, knee, and foot	Maintain joint mobility and prevent stiffness
Stretching	Achilles tendon stretching	Prevent equinus contracture
Muscle strengthening	Progressive strengthening of knee flexors/extensors, ankle dorsiflexors, and intrinsic foot muscles	Improve muscle strength and function
Proprioceptive training	Toe curls, marble picking, ball lifts, heel-to-toe rocking	Improve proprioception and motor control
Neuromuscular stimulation	Neuromuscular electrical stimulation (NMES) of dorsiflexor muscles	Reduce disuse atrophy and enhance motor activation
Gait training	Progressive gait training (parallel bars to independent ambulation)	Restore functional gait and balance
Home program	Video-guided home exercise program	Ensure continuity of rehabilitation and reinforce recovery

In addition, the patient received two ultrasound-guided platelet-rich plasma (PRP) injections, spaced four weeks apart, targeting the sciatic nerve and the denervated ankle dorsiflexor muscles. PRP was prepared from 20 mL of autologous venous blood collected into sodium citrate tubes and processed using a double-centrifugation protocol with a first spin at 1500 rpm for 10 minutes followed by a second spin at 2500 rpm for 10 minutes, yielding approximately 5 mL of platelet-rich plasma. No exogenous activation agent was used prior to injection. All injections were performed under real-time high-frequency ultrasound guidance using a linear probe at 12-15 MHz, with the patient in the prone position. At each session, 3 mL of PRP was administered perineurally around the sciatic nerve at the gluteal level, and an additional 2 mL was injected intramuscularly into the tibialis anterior muscle. The injections were well tolerated, and no local or systemic adverse events were reported.

After six months of follow-up, objective functional improvement was observed. Ankle dorsiflexion strength improved from MRC grade 1/5 at initial assessment to 4/5, allowing active foot clearance during gait [4]. The patient progressed from a steppage gait pattern to independent ambulation with the use of an ankle-foot orthosis. Knee and ankle joint mobility were preserved, and no equinus deformity developed. Mild residual sensory deficits persisted over the plantar surface.

## Discussion

Sciatic nerve injury in the context of acetabular fractures can occur through three main mechanisms: direct injury from the initial trauma, iatrogenic injury during surgery, or delayed postoperative injury [1]. Perioperative causes include excessive traction on the nerve, inappropriate retractor placement, and instrument or implant-related complications [1]. From that, the clinical history detailing the date of symptom onset relative to surgery and the physical examination data are both essential for identifying the correct etiology of the sciatic nerve injury. 

Prevention of intraoperative sciatic nerve injury relies on several well-established strategies. Letournel and Judet recommended maintaining hip extension with knee flexion when operating via the posterior approach to reduce tension on the sciatic nerve [5]. Careful retractor placement is equally important, as excessive posterior retraction with the hip in flexion significantly increases the risk of nerve traction injury [6]. Intraoperative neurophysiological monitoring using somatosensory evoked potentials (SSEP). Intraoperative spontaneous electromyography can alert the surgeon to potential nerve injury during the procedure [7].

Electrodiagnostic studies are fundamental in the evaluation of sciatic neuropathies, enabling precise lesion localization within the nerve, characterization of injury severity (axonotmesis vs. neurotmesis), and prognostic assessment of recovery potential [8].

A well-established finding in sciatic neuropathy is the preferential involvement of the peroneal division, which is consistently reported as more severely affected than the tibial division, likely due to its more superficial course and susceptibility to mechanical injury [9]. In a retrospective electrodiagnostic study of 109 patients with confirmed sciatic neuropathy, Cherian and Li found that predominant peroneal division involvement was present in 39.4% of cases, while tibial division predominance was rare (5.5%), and that sciatic neuropathies were axonal in nature in 95.4% of cases [10]. In our patient, both divisions were involved, with marked peroneal predominance and axonal injury pattern on electrodiagnostic testing, consistent with these reported findings.

Prognostic factors for functional recovery following sciatic nerve injury include the preservation of compound muscle action potentials (CMAPs) in distal muscles, such as the extensor digitorum brevis, and residual plantar flexion and dorsiflexion strength at initial assessment [3]. Absence of these responses indicates severe axonal loss and is associated with a less favorable prognosis. In our case, initial electrodiagnostic studies showed absent fibular motor responses, suggesting significant axonal injury, yet meaningful functional recovery was achieved within six months.

Spontaneous recovery following sciatic nerve injury associated with acetabular fractures has been reported in the literature. In a recent meta-analysis, Stavrakakis et al. reported complete recovery rates of 64.7% for post-traumatic injuries and 74.1% for iatrogenic injuries [2].

The use of platelet-rich plasma (PRP) as an adjunct treatment in peripheral nerve regeneration is supported by a growing body of experimental evidence. PRP contains a high concentration of growth factors, including PDGF (platelet-derived growth factor), VEGF (vascular endothelial growth factor), IGF-1 (insulin-like growth factor 1), and NGF (nerve growth factor), that are known to promote Schwann cell proliferation and axonal regrowth in tissues with inherently low regenerative capacity [11]. Animal models have consistently demonstrated enhanced nerve regeneration following PRP application [12,13]. Clinical reports, though limited in number, have shown encouraging outcomes following PRP treatment for peripheral nerve injuries [14-15]. In our case, ultrasound-guided perineural PRP injections were well tolerated and may have contributed to the observed recovery. However, given the known potential for spontaneous recovery in sciatic nerve injuries and the concomitant rehabilitation program, the specific contribution of PRP cannot be definitively established in this case. 

Structured rehabilitation plays a crucial role in functional recovery after peripheral nerve injury. Its primary goals include preventing secondary musculoskeletal complications (joint contractures, muscle atrophy), promoting nerve regeneration through appropriate sensorimotor stimulation, restoring functional independence in gait and activities of daily living, and providing compensatory strategies during the nerve recovery period. Early prescription of an ankle-foot orthosis (AFO) is recommended to prevent equinus deformity and to safely restore gait mechanics during the denervation period, as it maintains the ankle in neutral position during the swing phase and reduces fall risk [16]. Neuromuscular electrical stimulation (NMES) applied to denervated muscles is used to retard disuse atrophy, maintain muscle fiber integrity, and facilitate motor relearning when reinnervation begins, and has demonstrated benefit in foot drop rehabilitation across multiple neurological conditions [17].

In our case, combining multimodal rehabilitation with ultrasound-guided PRP injections led to meaningful functional recovery within six months, despite an initially unfavorable electrodiagnostic profile with absent peroneal motor responses. In a large surgical series of 380 patients followed over 24 years, Kline et al. confirmed that peroneal division injuries carried a substantially worse prognosis than tibial division injuries, with only 36% achieving significant recovery after surgical repair [18]. However, this comparison should be interpreted with caution due to differences in injury severity and patient populations. In contrast to these surgical outcomes, our patient achieved significant functional recovery through a non-surgical multimodal approach combining early rehabilitation and PRP injections.

## Conclusions

Sciatic nerve injury associated with acetabular fractures represents a serious complication that can arise from traumatic, perioperative, or postoperative mechanisms. A systematic clinical and electrodiagnostic evaluation is essential to establish the etiology, characterize the lesion, and guide treatment. Preventive intraoperative measures, including optimal patient positioning, careful retractor placement, and neurophysiological monitoring, should be systematically implemented. Early multimodal rehabilitation, combined with emerging adjunctive therapies such as ultrasound-guided PRP injections, may optimize functional recovery as observed in this case despite unfavorable initial electrodiagnostic features. However, randomized controlled trials and larger case series are needed to validate the role of PRP in the treatment of sciatic nerve injury.
